# Patient‐derived xenograft models of ALK+ ALCL reveal preclinical promise for therapy with brigatinib

**DOI:** 10.1111/bjh.18953

**Published:** 2023-06-25

**Authors:** Nina Prokoph, Jamie D. Matthews, Ricky M. Trigg, Ivonne A. Montes‐Mojarro, G. A. Amos Burke, Falko Fend, Olaf Merkel, Lukas Kenner, Birgit Geoerger, Robert Johnston, Matthew J. Murray, Charlotte Riguad, Laurence Brugières, Suzanne D. Turner

**Affiliations:** ^1^ Division of Cellular and Molecular Pathology, Department of Pathology University of Cambridge, Addenbrooke's Hospital Cambridge UK; ^2^ Institute of Pathology and Neuropathology and Comprehensive Cancer Center Tübingen University Hospital Tübingen, Eberhard‐Karls‐University Tübingen Germany; ^3^ Department of Paediatric Haematology and Oncology Cambridge University Hospitals NHS Foundation Trust Cambridge UK; ^4^ Department of Experimental Pathology and Laboratory Animal Pathology, Institute of Clinical Pathology Medical University of Vienna Vienna Austria; ^5^ Unit of Laboratory Animal Pathology University of Veterinary Medicine Vienna Vienna Austria; ^6^ Christian Doppler Laboratory for Applied Metabolomics Medical University of Vienna Vienna Austria; ^7^ Center for Biomarker Research in Medicine (CBmed) Vienna, Core‐Lab2 Medical University of Vienna Vienna Austria; ^8^ Department of Pediatric and Adolescent Oncology Gustave Roussy Cancer Center Villejuif France; ^9^ INSERM U1015, Gustave Roussy Cancer Center Université Paris‐Saclay Villejuif France; ^10^ Department of Paediatric Oncology/Haematology Royal Belfast Hospital for Sick Children Belfast UK; ^11^ Institute of Medical Genetics and Genomics, Faculty of Medicine Masaryk University Brno Czech Republic

**Keywords:** ALCL, brigatinib, crizotinib, PDX, tyrosine kinase inhibitors

## Abstract

Anaplastic large‐cell lymphoma (ALCL) is a T‐cell malignancy predominantly driven by the oncogenic anaplastic lymphoma kinase (ALK), accounting for approximately 15% of all paediatric non‐Hodgkin lymphoma. Patients with central nervous system (CNS) relapse are particularly difficult to treat with a 3‐year overall survival of 49% and a median survival of 23.5 months. The second‐generation ALK inhibitor brigatinib shows superior penetration of the blood–brain barrier unlike the first‐generation drug crizotinib and has shown promising results in ALK+ non‐small‐cell lung cancer. However, the benefits of brigatinib in treating aggressive paediatric ALK+ ALCL are largely unknown. We established a patient‐derived xenograft (PDX) resource from ALK+ ALCL patients at or before CNS relapse serving as models to facilitate the development of future therapies. We show in vivo that brigatinib is effective in inducing the remission of PDX models of crizotinib‐resistant (ALK C1156Y, *TP53* loss) ALCL and furthermore that it is superior to crizotinib as a second‐line approach to the treatment of a standard chemotherapy relapsed/refractory ALCL PDX pointing to brigatinib as a future therapeutic option.

## Graphical abstract

Patient derived xenograft models of crizotinib‐resistant ALK+ ALCL developed from patients with CNS involvement are responsive to brigatinib despite harbouring mutations in ALK and TP53

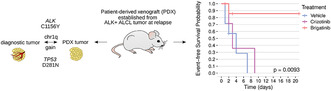

## INTRODUCTION

Systemic anaplastic large‐cell lymphoma (ALCL) is a T‐cell malignancy accounting for approximately 15% of all paediatric non‐Hodgkin lymphoma (NHL).^1^ More than 90% of paediatric cases express anaplastic lymphoma kinase (ALK) fusion proteins (ALK+) as a result of translocations, the most predominant being the t(2;5)(p23;q35) encoding Nucleophosmin 1 (NPM)‐ALK.^2^, ^3^ While for most patients, ALCL99 therapy leads to a good outcome with the 10‐year overall survival (OS) reaching 90%, progression‐free survival (PFS) at approximately 70% requires improvement.^4^ Recent addition of the first‐generation ALK tyrosine kinase inhibitor (TKI), crizotinib to the standard ALCL99 backbone gives a similar OS of 95%, but this reflects just 2 years of follow‐up at which point event‐free survival (EFS) is 76.8%, suggesting that the addition of crizotinib protects against relapse at this early stage.^5^

At present, 85% of patients treated with a standard chemotherapy backbone who relapse after completion of front‐line therapy enter a second remission regardless of the chemotherapy regimen used.^6^, ^7^, ^8^ However, approximately 50% of children who progress during front‐line therapy will experience progression during reinduction.^8^ For paediatric ALK+ ALCL patients that relapse from chemotherapy, crizotinib has been trialled as a salvage therapy (NCT00939770, NCT01606878, NCT01979536, NCT02304809, UMIN000028075, Eudract: 2015‐005437‐53)^9^, ^10^, ^11^, ^12^, ^13^ with the aim to induce second remission^8^ and in some cases leading to allogeneic stem cell transplant (SCT) as established for adult patients with relapsed ALK+ ALCL.^14^, ^15^ For patients who relapse in the central nervous system (CNS), the 3‐year OS is only 48.70%.^16^ Brigatinib, which has good CNS penetration, is now being investigated for relapsed/refractory (r/r) ALK+ ALCL (NCT04925609), and in studies of ALK+ NSCLC, it is effective in patients who have failed crizotinib.^17^

Here, we describe three PDXs of ALK+ ALCL developed from patients who all had CNS disease or CNS relapse, comprising a subgroup of patients with unmet clinical needs.^18^, ^19^ We demonstrate that these models recapitulate the biology of parental human tumours and that the PDX platform serves as a tool for the discovery and testing of targeted therapies. Specifically, we demonstrate the effectiveness of brigatinib in these PDXs in the context of potential crizotinib resistance mechanisms, including *ALK* copy gain, *ALK* C1156Y mutation and *IRF4* amplification.

## MATERIALS AND METHODS

Information about PDX establishment, animal licencing, Whole Exome Sequencing, immunohistochemistry and ethics approvals can be found in the Supporting Information with reagents and resources detailed in Table S1.

### In vivo studies

Viably frozen PDX cells were thawed, washed in PBS/2% FBS before resuspension in Matrigel:PBS (1:2) and subcutaneous injection of 0.5 × 10^6^ cells into the left flank of a NOD./Cg‐Prkdc^scid^Il2rg^tm1Wjl^/SzJ mouse. Once tumours reached 400 mm^3^, mice were randomly assigned to one of three treatment groups and treated daily by oral gavage with either vehicle (1 × PBS, 10% DMSO), 100 mg/kg crizotinib or 25 mg/kg brigatinib.

### Sequencing data analysis

Quality control, processing and variant calling steps were undertaken as part of an established in‐house, publicly available Snakemake pipeline,^20^ with software deployed in isolated Conda environments and executed in a versioned Singularity container. Briefly, FastQC was used to assess sequence‐level quality before Xengsort was employed to filter murine host reads in silico prior to alignment to the human genome (GRCh38.p14) with bwa mem. Small nucleotide variants were called using Octopus and Mutect2. Input CRAM files for Mutect2 were processed according to the GATK Best Practices preprocessing guidelines.^21^ Structural variants were called from WES using DELLY, and gene fusions were called from RNAseq data using STAR‐Fusion. Allele‐specific copy number calls and purity estimates were generated for the tumour sample from Patient 3 and corresponding GR‐ALCL‐1 PDX using sequenza (v3.0.0), using a bin width of 50. Variants at the nucleotide level are reported on the canonical transcript, according to Ensembl.

### Statistical analysis

All statistics were calculated using R (v4.0.2). Benjamini–Hochberg adjusted *p*‐values were calculated with the logrank_test function from the coin package (v1.4‐1) and adjusted with the stats::p.adjust function setting method = ‘BH’. Mouse weight data were analysed by fitting a linear model (stats::lm) to the weight difference from baseline to the end of the study, with baseline weight and treatment used as predictors.

## RESULTS

### Established PDX models maintain histologic features of the engrafted ALK+ ALCL tumours

**FIGURE 1:**
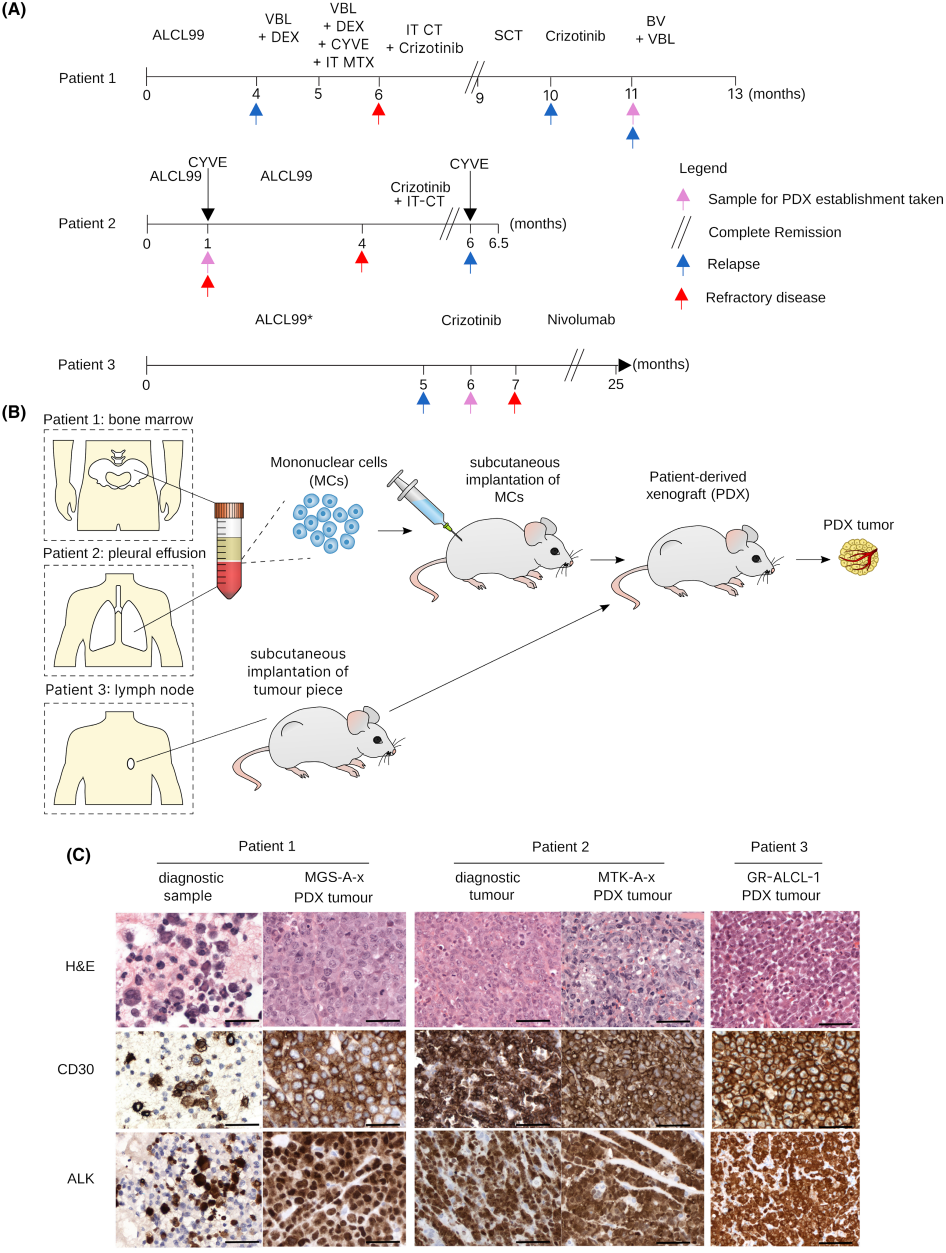
Established PDX models maintain histologic features of the engrafted ALK+ ALCL tumours. (A) Schema of the treatment history of ALK+ ALCL patients. (B) Schema of PDX generation; mononuclear cells (MCs) were isolated from a bone marrow (Patient 1), pleural effusion (Patient 2) or paravertebral tumour lymph node (Patient 3) sample and injected subcutaneously into NSG mice to establish PDX models of ALK+ ALCL. (C) Representative haematoxylin and eosin staining (400×) with corresponding ALK and CD30 immunohistochemistry (400×) performed on sections of the pleural effusion taken at diagnosis (Patient 1), diagnostic tumour (Patient 2) compared with the PDX (at passage 3). No material was available for Patient 3, and the PDX alone is shown. BV, brentuximab vedotin; SCT, stem cell transplant; VBL, vinblastine; CYVE, cytarabine + etoposide. See Table S2 for further patient details. [Colour figure can be viewed at wileyonlinelibrary.com]

All PDX models are derived from patients who experienced r/r disease during front‐line ALCL99 chemotherapy^4^ (Figure 1A, Table S2). Patient 1 (MGS‐A‐x) further progressed with CNS involvement while undergoing treatment with vinblastine combined with intravenous and intrathecal chemotherapy. Since this treatment was poorly tolerated and only an incomplete response was achieved, the patient commenced crizotinib alongside intrathecal chemotherapy, achieving a CR. The patient then received an allogeneic SCT but progressed rapidly thereafter. The patient was re‐treated with crizotinib until eventual progression. Mononuclear cells (MCs) isolated from a bone marrow sample at this timepoint were used to generate the PDX (Figure 1B). Despite subsequent treatment with weekly brentuximab vedotin and vinblastine, the patient died due to progressive disease and the complications of therapy shortly after.

Patient 2 (MTK‐A‐x) commenced crizotinib treatment with intrathecal chemotherapy due to continued refractory disease, despite treatment including ALCL99 chemotherapy (Figure 1A, Table S2). MCs were isolated from a pleural effusion obtained early in the disease course, before crizotinib initiation, and were used to generate the PDX (Figure 1B). Despite an excellent initial response to crizotinib, with CR confirmed on imaging 7 weeks after initiation, Patient 2 relapsed with aggressive isolated CNS involvement shortly afterwards and died despite further intensive conventional intravenous chemotherapy.

Patient 3 (GR‐ALCL‐1) was treated according to ALCL99 protocol recommendations for patients with CNS involvement achieving partial response after the first three chemotherapy cycles but relapsed while still on chemotherapy.^22^ Crizotinib treatment was then commenced, but disease progression led to the cessation of treatment 2 months after initiation (Figure 1A, Table S2). A paravertebral lymph node was sampled 1 month after crizotinib initiation, and this was used to establish the PDX (Figure 1B)^23^. This patient achieved a CR following subsequent treatment with nivolumab and remains in CR 5 years later.

Tumour growth in mice was observed within 6 months of implantation for all three patients; tumours were confirmed by immunohistochemistry to be positive for ALK and CD30 expression at passage 3 (Figure 1C). Histologic concordance was noted between the PDXs and primary tumours for cases with available corresponding diagnostic biopsy material (Figure 1C).

### The genomic profile of the implanted ALK+ ALCL tumour is maintained in the PDX

To determine whether the established PDX preserves the genomic profile of the implanted tumour, DNA was isolated from peripheral blood mononuclear cells (PBMCs) and the relapse tumour biopsy from Patient 3, as well as the corresponding established PDX at passage 4.

**FIGURE 2:**
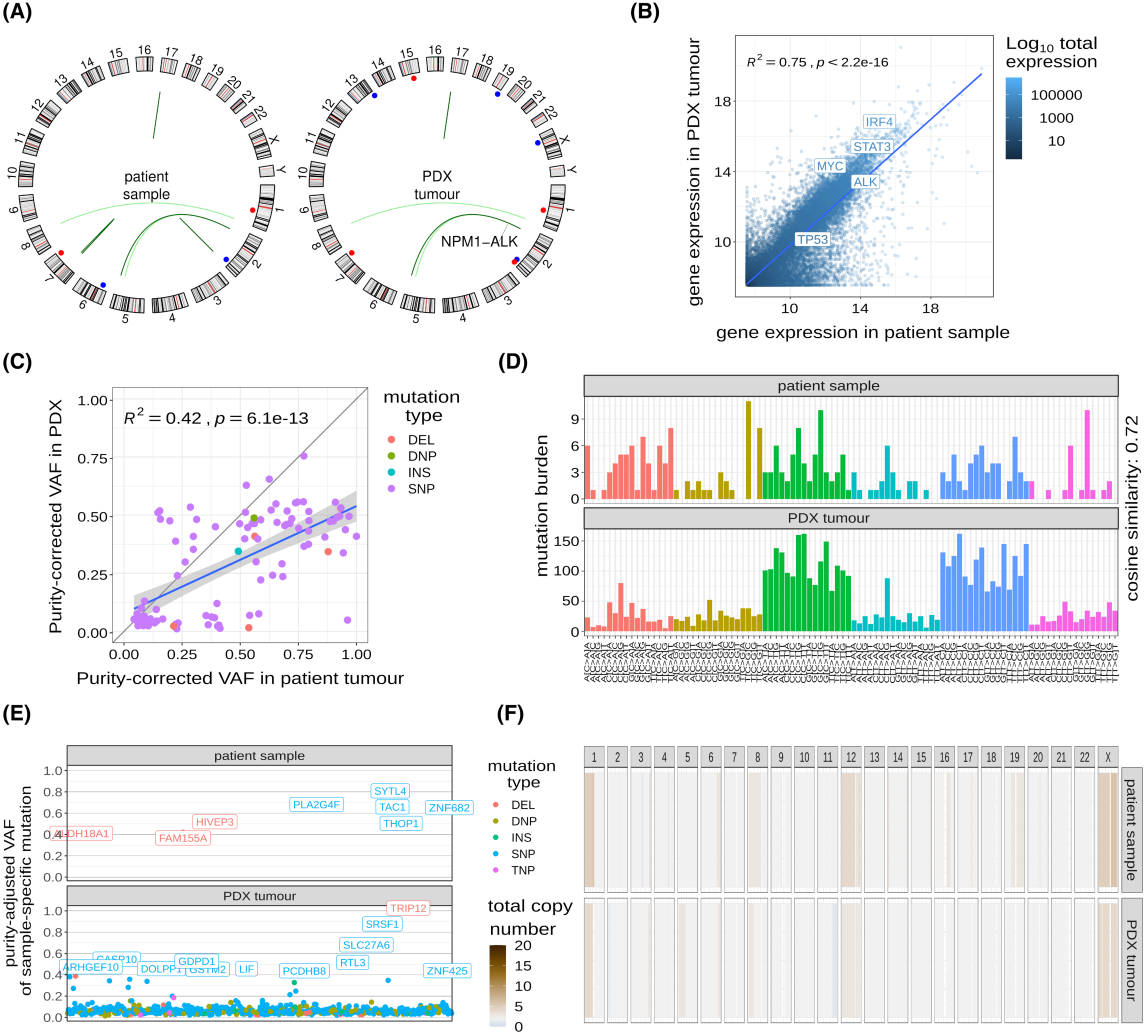
The genomic profile of the implanted ALK+ ALCL tumour is maintained in the PDX. (A) Circos representation of structural variants detected from WES and RNAseq data for the patient sample (left plot) and the passage 4 PDX sample (right plot). Blue points represent insertions, while red points represent deletions. Arcs represent translocations. (B) Scatter plot showing normalised gene expression (transcripts per million) for the tumour sample from Patient 3 and the derived PDX tumour (GR‐ALCL‐1; passage 4). The blue line represents the linear regression fit. (C) Scatter plot showing the joint empirical VAF distribution of SNVs detected in both the tumour sample from Patient 3 and the derived PDX tumour (GR‐ALCL‐1; passage 4). The blue line represents the linear regression fit. (D) Mutational catalogues showing the burden of SNVs according to the original and mutated bases, as well as the bases immediately 5′ or 3′ to the mutated base. (E) VAF of mutations detected that are unique to the patient sample or derived PDX. Mutations with a VAF greater than 0.2 are highlighted. (F) Total copy number profiles for the autosomes and X chromosome. Genomic segments are coloured according to the estimated total copy number. DEL, deletion; DNP, dinucleotide polymorphism; INS, insertion; R, correlation coefficient; SNP, single nucleotide polymorphism; TNP, trinucleotide polymorphism; VAF, variant allele frequency. [Colour figure can be viewed at wileyonlinelibrary.com]

As expected, the t(2;5) encoding NPM1‐ALK^24^ was detected in both the relapse biopsy and the established PDX (Figure 2A). Furthermore, structural variants, including a deletion on chr1 including the *IQGAP3* locus, a duplication on chr2 including *IDH1* and a breakend between *KIF26B* and *LRP12* (chr1 and chr8), were also detected in both the relapse biopsy and established PDX (Figure 2A). One duplication and two breakends detected in the relapse biopsy were not retained in the PDX; a duplication on chr6 involving the *EHMT2‐AS1* anti‐sense RNA gene, a gene fusion between *SEPTIN7P2* and *PSPH* on chr7 and a gene fusion between *TRIP12* and *DNER* on chr2 respectively (Figure 2A). Conversely, new structural variants were seen in the PDX but not in the original biopsy, including the deletion of a region containing *ANXA2*, as well as duplication of regions containing *RAB20*, *COL4A2*, *FIZ1* and *DDX3X* (Figure 2A).

Despite differences in some structural variants, the transcriptomes of the samples demonstrated concordance, with 75% of the gene expression variance in the PDX tumour explained by gene expression in the patient sample (*R* ^2^ = 0.75, *p* < 2.2e‐16; Figure 2B). In particular, key genes previously associated with the biology of ALCL including *STAT3* were expressed in both the PDX and primary tumour.^25^

Next, subclonal SNVs were assessed by comparing variant allele frequencies (VAF) for those detected before and after tumour engraftment in the mouse (Figure 2C, Figure S1). While many SNVs lie off the scatterplot diagonal, indicating clonal selection, VAFs across the two samples highly correlated, with 42% of the VAF variance in the PDX explained by VAF in the patient tumour (Figure 2D). Furthermore, considering the trinucleotide context, the mutational catalogue of the patient tumour sample was well‐preserved in the PDX (cosine similarity 0.72; Figure 2D).

The majority of small variants detected in the PDX but not the primary tumour were detected at a low VAF (<0.2), consistent with the ongoing accumulation of mutations between or during cell division (Figure 2E). However, several small variants that were not detected in the patient sample attained high frequency (>40%) in the PDX, including a deletion in *TRIP12* suggesting ongoing clonal evolution in vivo (Figure 2E).

Copy number alterations were largely maintained in the PDX, though some events including del(11q) and gain(5q) were detected in the PDX but not the patient sample, while events including gain(19q) appeared to be lost in the PDX tumour (Figure 2F). Together, these data show that although there is ongoing clonal evolution upon xenotransplantation, the PDX maintains the landscape of somatic mutations and oncogenic drivers displayed in its parental ALK+ ALCL tumour.

### Established PDX models retain the crizotinib sensitivity of the engrafted ALK+ ALCL tumour and are sensitive to brigatinib

**FIGURE 3:**
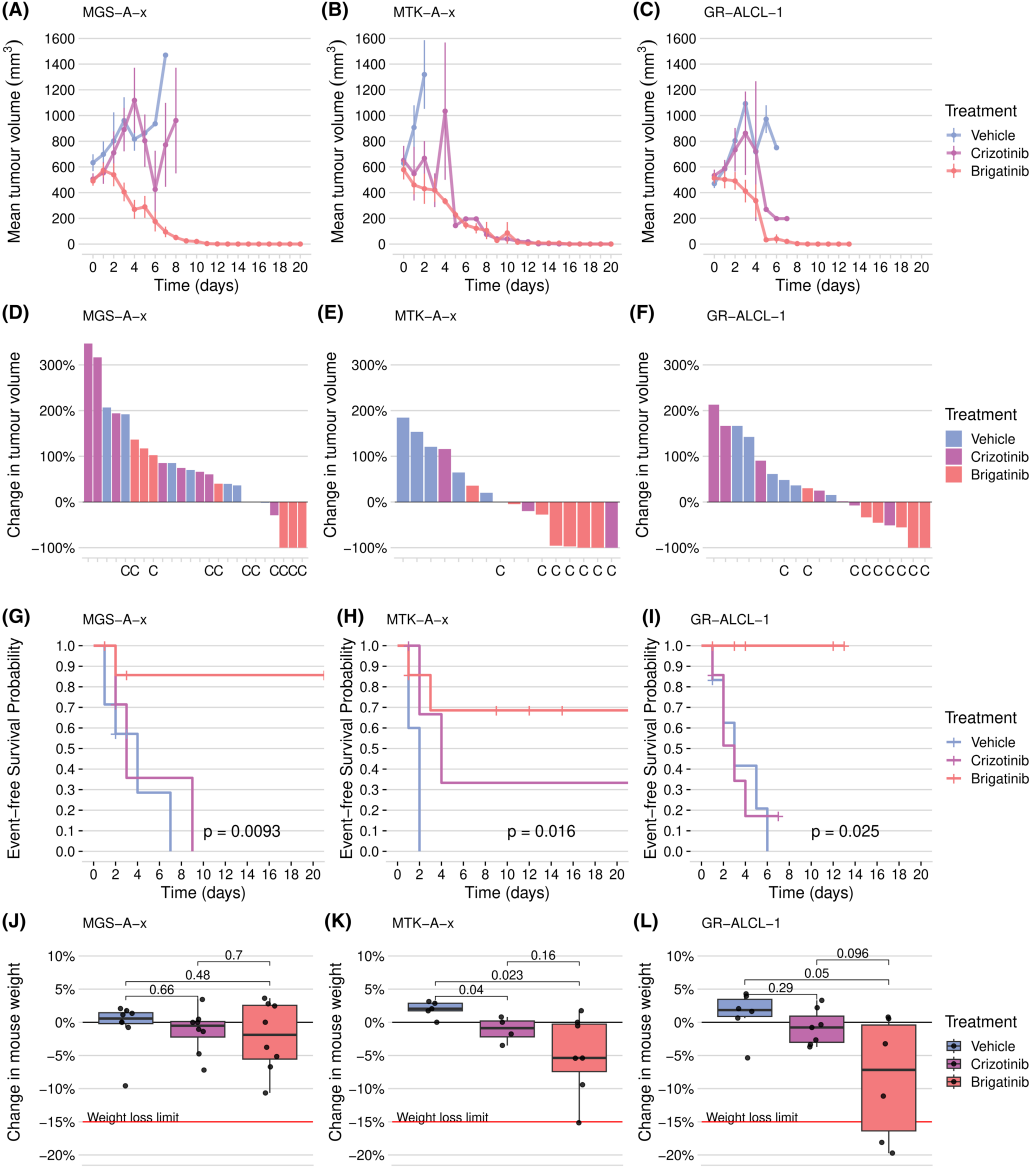
Established PDX models retain the chemotherapy sensitivity of the engrafted ALK+ ALCL tumours and are sensitive to brigatinib. (A–C) Tumour volume over time for MGS‐A‐x (A), MTS‐A‐x (B) and GR‐ALCL‐1 (C) mice administered vehicle (PBS, 10% DMSO), crizotinib (100 mg/kg) or brigatinib (25 mg/kg) daily by oral gavage. Data represent the mean and standard error of each treatment group (crizotinib: four mice MTX‐A‐x, eight mice MGS‐A‐x, six mice GR‐ALCL‐1; Vehicle: five mice MTX‐A‐x, six mice GR‐ALCL‐1; brigatinib: eight mice for all treatment groups). Day 0 refers to the day that the tumour volume first met or exceeded 400 mm^3^ (baseline) and treatment was initiated. Individual mouse tumour measurements were included until the mouse either reached the event (defined as the tumour reaching or exceeding 15 mm in any direction) or was censored (removed from the study due to reaching a humane end‐point other than tumour size, or 21 consecutive days of treatment). (D–F) Percentage change in tumour volume at the study end‐point compared with the baseline volume for individual tumour‐bearing MGS‐A‐x (D), MTK‐A‐x (E) or GR‐ALCL‐1 (F) mice. (G–I) Kaplan–Meier EFS for tumour‐bearing MGS‐A‐x (G), MTS‐A‐x (H) or GR‐ALCL‐1 (I) mice, where an event is defined as the tumour reaching or exceeding 15 mm in any direction. Censoring is indicated by vertical ticks. The *p*‐value is determined by the log‐rank test. (J–L) Mouse body weights at the experimental end‐point relative to baseline weights for MGS‐A‐x (G), MTS‐A‐x (H) or GR‐ALCL‐1 (I) mice. *p*‐values were determined by pairwise two‐sample *t*‐tests. [Colour figure can be viewed at wileyonlinelibrary.com]

To determine whether the PDX tumours retain their sensitivity or resistance to crizotinib as seen in the patients (Figure 1A), mice were exposed to the drug (100 mg/kg) or vehicle (1 × PBS, 10% DMSO) once tumours reached 400 mm^3^ in volume (Figure 3, Figure S2). As expected, the MTK‐A‐x PDX derived from Patient 2 at a time when they were naïve to crizotinib showed a significant increase in EFS for animals treated with crizotinib relative to vehicle (*p* = 0.048, Figure 3H). While 3/4 mice that were treated with crizotinib showed a reduction in tumour volume and 1/4 mice demonstrated a complete response, 5/5 mice that were treated with vehicle presented with tumour progression (Figure 3E) within 1 day after treatment initiation (Figure 3B).

The other two PDXs (MGS‐A‐x and GR‐ALCL‐1) were developed from tumour samples taken from Patients 1 and 3, respectively, when they had become r/r to crizotinib (Figure 1A, Table S2). As expected, both were resistant to crizotinib, with the tumours continuing to grow despite treatment. For MGS‐A‐x, 7/8 mice showed tumour progression (Figure 3D) with no significant difference in EFS for animals treated with crizotinib compared with the vehicle (*p* = 0.4303; Figure 3G). Similarly, the GR‐ALCL‐1 PDX model was also resistant to crizotinib whereby 6/6 and 4/6 mice treated with vehicle or crizotinib, respectively, presented with tumour progression (Figure 3F) within 2 days after treatment initiation (Figure 3C). Again, EFS did not significantly differ for animals treated with crizotinib or vehicle (*p* = 0.9779; Figure 3I).

The response of all three PDXs to an alternative ALK inhibitor, brigatinib, was assessed by exposing mice to 25 mg/kg of the drug. Brigatinib was chosen for study here as it is currently the subject of a European trial for r/r ALK+ ALCL (NCT04925609) and has good brain penetration. For all three PDXs, significant increases in EFS were seen for tumour‐bearing animals treated with brigatinib relative to vehicle (MGS‐A‐x: *p* = 0.02494, MTK‐A‐x: *p* = 0.048, GR‐ALCL‐1: *p* = 0.01299; Figure 3G–I), with 3/8, 5/8 and 5/8 mice, respectively, showing a reduction in tumour volume (Figure 3D–F). Brigatinib treatment also led to a significant increase in EFS relative to crizotinib‐treated mice for MGS‐A‐x and GR‐ALCL‐1, but not for MTK‐A‐x (MGS‐A‐x: *p* = 0.02494, MTK‐A‐x: *p* = 0.58, GR‐ALCL‐1: *p* = 0.01923; Figure 3G–I). Brigatinib was generally well‐tolerated, though significant decreases in animal weight relative to vehicle‐treated mice were detected for MTK‐A‐x‐ and GR‐ALCL‐1‐bearing mice (MTK‐A‐x: *p* = 0.023, GR‐ALCL‐1: *p* = 0.05; Figure 3K,L). In comparison, crizotinib led to a significant decrease in animal weight relative to vehicle‐treated mice for MTK‐A‐x‐bearing mice only (*p* = 0.04; Figure 3K). Altogether, these data suggest that brigatinib administration is a well‐tolerated approach for the treatment of PDX derived from ALK‐positive ALCL involving the CNS that is either sensitive or r/r to crizotinib.

### *ALK* C1156Y mutation, *TP53* loss and *IRF4* amplification are likely mediators of resistance to crizotinib

**FIGURE 4:**
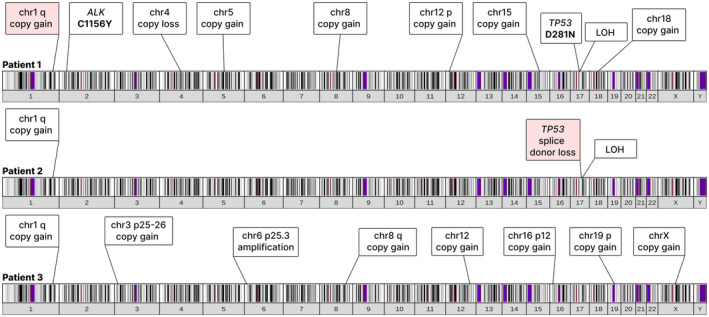
ALK C1156Y mutation, TP53 loss and IRF4 amplification are likely mediators of resistance to crizotinib. WES data determined from the established PDX were analysed for Patients 1–3. Events highlighted in pink were also detected in the corresponding diagnostic biopsies for Patients 1 and 2; diagnostic material was not available for comparisons to be made for Patient 3. The banding pattern represents cytoband staining. [Colour figure can be viewed at wileyonlinelibrary.com]

For Patients 1 and 2, the corresponding diagnostic tumour was available for WES enabling the comparison of the mutational profiles of cells at diagnosis and the corresponding PDXs derived from the relapse samples (Figure 4). We focussed on large‐scale alterations as germline samples were not available for these patients.

The PDX of Patient 1 (MGS‐A‐x) harbours an *ALK* c.3467G>A mutation (33% VAF) not present in the diagnostic biopsy (Figure 4). This mutation, corresponding to C1156Y in full‐length ALK, is near the αC helix domain and was first described in a crizotinib‐resistant ALK+ NSCLC patient^26^ causing resistance via conformational changes that alter kinase activity in line with in vivo results (Figure 3G).^27^ In addition to the *ALK* mutation, single copy number gains were detected at the *NPM1* and *ALK* loci, suggesting that copy gain of *NPM1‐ALK* occurred first, followed by the *ALK* c.3467G>A mutation occurring on one of the two translocated copies of *ALK*. Moreover, a heterozygous *TP53* c.841G>A mutation was present in the PDX (Figure 4). This mutation has previously been described to inactivate p53 in a dominant‐negative fashion^28^, ^29^ and was present at a frequency of 100% in the PDX, suggesting that the wild‐type allele was lost some time between diagnosis and passage 4 (Figure 4). In addition, copy number changes were detected in the PDX but not the diagnostic biopsy, including copy loss of chr4, and copy gains of chr5, chr8, chr15, chr18 and gain(12p) (Figure 4), while gain(1q) was present in both the diagnostic biopsy and PDX.

In contrast to Patient 1, no mutations or copy number alterations of NPM1‐ALK were detected in the relapse biopsy nor the derived PDX of Patient 3 (GR‐ALCL‐1), despite the noted crizotinib resistance (Figure 3C,F,I). However, there was an amplification of *IRF4* in the implanted tumour material, though diagnostic tumour material was not available to rule out its presence prior to relapse. However, a previously published CRISPRa screen showed that IRF4 overexpression mediates crizotinib resistance in ALK+ ALCL cell lines,^30^ supporting its role in driving crizotinib resistance in the patient. Several additional copy number gains were also detected in the relapse biopsy, including gain(1q) and gain(8q), which have also previously been associated with ALK+ ALCL^31^, ^32^, ^33^ (Figure 4, Figure S3).

Similarly, and as expected, no mutations or copy number alterations of NPM1‐ALK were detected in the diagnostic sample nor PDX derived from Patient 2 (MTK‐A‐x), in line with a lack of clinical resistance to crizotinib, and the PDX being established from a crizotinib‐naïve patient sample. Interestingly, a *TP53* mutation in the intron 6 splice donor site was detected in the diagnostic sample of Patient 2 (Figure 4). This variant has previously been described to activate a cryptic splice site in intron 6, resulting in loss of function.^34^ Similar to Patient 1, sometime between diagnosis and passage 4 of the PDX, the *TP53* locus underwent loss of heterozygosity, likely resulting in biallelic loss of p53 function. No large‐scale copy number alterations were detected in the diagnostic sample, but the PDX harboured a gain(1q) (Figure 4). Given the complex treatment history of this patient (Figure 1A), these changes may be related to exposure to chemotherapeutic agents.

## DISCUSSION

We have successfully established and characterised PDX models from biopsies of multi‐agent chemotherapy‐refractory (Patient 2, MTK‐A‐x), and both multi‐agent chemotherapy‐refractory‐ and crizotinib‐resistant (Patient 1, MGS‐A‐x; Patient 3, GR‐ALCL‐1) paediatric CNS‐positive, r/r ALK+ ALCL patients. These PDX models retain the drug sensitivity of the engrafted patient tumour.

Our in vivo investigation shows that brigatinib is effective in PDX models developed from CNS‐relapsed chemotherapy‐refractory and/or crizotinib‐resistant ALK+ ALCL patients. The advantage of brigatinib, alectinib and lorlatinib over crizotinib is that they have a superior ability to cross the blood–brain barrier (BBB) and as such are active or preventive against CNS disease.^35^, ^36^, ^37^, ^38^, ^39^ Indeed, the use of these inhibitors has led to CR for several published r/r ALK+ ALCL cases with CNS involvement.^40^ Hence, while our models do not account for the CNS location of the relapse disease, it can be assumed that as these drugs are better able to cross the BBB, they will inhibit tumour growth and/or disease progression and as such might also represent better therapeutic options for the up‐front treatment of CNS‐positive ALCL. However, this will require validation in a clinical trial setting.

Analysis of the established PDX has also allowed us to postulate mechanisms of resistance to ALK inhibitors. In particular, the PDX of Patient 1 (MGS‐A‐x) carries an ALK C1156Y mutant previously reported to mediate resistance to crizotinib, but not brigatinib.^27^ This phenotype was maintained in the PDX. Unfortunately, diagnostic biopsy material was not available for this patient, and so, we are unable to determine whether the responsible mutation was present in a minor subclone of tumour cells at diagnosis that was selected with crizotinib treatment. Furthermore, our data suggest that brigatinib may be a potential therapeutic option for this patient.

For Patient 3, the engrafted tumour and the relapse biopsy both demonstrated amplification and high expression of IRF4. IRF4 has previously been shown to be overexpressed in several NHLs, such as cutaneous ALCL,^41^ and notably large B‐cell lymphoma with IRF4 rearrangement.^42^ Indeed, IRF4 knockdown induces apoptosis in both ALK+ and ALK‐ALCL cell lines, and ectopic expression of IRF4 in ALCL cell lines has been shown to partially rescue STAT3 knockdown.^43^, ^44^ IRF4 may also constitute an ALK inhibitor bypass resistance track as previously suggested in a CRISPRa screen of ALCL.^30^ However, why IRF4 amplification would mediate resistance to crizotinib, but not brigatinib, is not clear.

Finally, all three of these aggressive cases of CNS‐positive ALK+ ALCL were assessed for genomic events in the established PDX developed from r/r tissue. Given the intensive chemotherapy regimens experienced by all of the patients, a complex genome is expected. In this regard, a *TP53* mutation in the first and second PDX is in keeping with a poor prognosis^33^ and multiple regions of genomic gain and loss are consistent with r/r disease.^45^

In conclusion, we have developed PDX models derived from CNS‐involved ALCL that demonstrate sensitivity to brigatinib, even when known crizotinib resistance mechanisms are active.

## AUTHOR CONTRIBUTIONS

Nina Prokoph, Jamie D. Matthews and Suzanne D. Turner involved in conceptualization and methodology; Nina Prokoph, Jamie D. Matthews, Ricky M. Trigg and Ivonne A. Montes‐Mojarro involved in investigation; Nina Prokoph, Jamie D. Matthews and Ricky M. Trigg involved in visualisation; Nina Prokoph involved in writing—original draft; Jamie D. Matthews, Ricky M. Trigg, Matthew J. Murray and Suzanne D. Turner involved in writing—review and editing; all authors involved in writing—final approval; Birgit Geoerger and Suzanne D. Turner involved in funding acquisition; Birgit Geoerger, Laurence Brugières, Matthew J. Murray, Lukas Kenner and Robert Johnston involved in resources.

## FUNDING INFORMATION

This work was supported by the Cancer Research UK Cambridge Centre [C9685/A25117]. N.P., I.A.M.M, L.K. and S.D.T. were supported by a European Union Horizon 2020 Marie Skłodowska‐Curie Innovative Training Network Grant, grant no. 675712; J.D.M. by the Alex Hulme Foundation; S.D.T. was supported by the project National Institute for Cancer Research (Programme EXCELES, ID Project No. LX22NPO5102)—Funded by the European Union—Next Generation EU; L.K. was supported by the COMET Competence Center CBmed—Center for Biomarker Research in Medicine (n. FA791A0906.FFG), and the module project microOne as well as the Christian‐Doppler Lab for Applied Metabolomics, and by the Austrian Science Fund (grants FWF: P26011, P29251 and P 34781); the MAPPYACTS trial was supported by the Institut National du Cancer grant PHRC‐K14‐175, the Foundation ARC grant MAPY201501241 and the Association Imagine for Margo; MAPPYACTS PDX development was supported by Fédération Enfants Cancers et Santé, Société Française de lutte contre les Cancers et les leucémies de l'Enfant et l'adolescent (SFCE), Association AREMIG and Thibault BRIET; Parrainage médecin‐chercheur of Gustave Roussy. This research was supported by the NIHR Cambridge Biomedical Research Centre (BRC‐1215‐20 014); the views expressed are those of the authors and not necessarily those of the NIHR or the Department of Health and Social Care.

## CONFLICT OF INTEREST STATEMENT

The authors have no conflicts of interest to declare.

## ETHICS STATEMENT

The study was approved by the Huntington research ethics committee (no. 07/Q0104/16). Patient 3 was included in the MAPPYACTS trial (ClinicalTrials.gov Identifier: NCT02613962). The MAPPYACTS trial protocol, amendments and informed consents were approved by the ethics committee and complied with local regulations and the Declaration of Helsinki (no. 2015‐A00464‐45).

## PATIENT CONSENT STATEMENT

Written informed parental consent was obtained according to the Declaration of Helsinki.

## CLINICAL TRIAL REGISTRATION (INCLUDING TRIAL NUMBER)

Patient 3 was included in the MAPPYACTS trial (ClinicalTrials.gov Identifier: NCT02613962).

## Supporting information


Data S1.

## Data Availability

Requests for data should be made to Prof Suzanne Turner (sdt36@cam.ac.uk).
