# Supplementary material for: Patient‐derived xenograft models of ALK+ ALCL reveal preclinical promise for therapy with brigatinib
Source: Br J Haematol. 2023 Jun 25;202(5):985–94. doi: 10.1111/bjh.18953 (PMC10952693; doi:10.1111/bjh.18953)
Supplement: Supplementary file 1 — Data S1. [file BJH-202-985-s001.docx]

## Supporting Information

## Supplementary Methods

See Table S2 for details of reagents and resources used.

### Patient samples

Formalin-fixed paraffin-embedded (FFPE) tissues, bone marrow, pleural effusion, peripheral blood and related clinical information from both male (n = 2) and female (n = 1) paediatric patients with ALK+ ALCL were obtained after written informed parental consent and according to the Declaration of Helsinki. The study was approved by the Huntington research ethics committee (no. 07/Q0104/16). Patient 3 was included in the MAPPYACTS trial (ClinicalTrials.gov Identifier: NCT02613962). The MAPPYACTS trial protocol, amendments, and informed consents were approved by the ethics committee and complied with local regulations and the Declaration of Helsinki (no. 2015-A00464-45).

### Patient derived xenograft establishment

Animal work was carried out under UK Home Office project licence number P4DBEFF63 according to the Animals (Scientific Procedures) Act 1986, was approved by the University of Cambridge Animal Welfare and Ethical Review Body (AWERB) and complied with all relevant ethical regulations for animal testing and research in the UK.

To establish MGS-A-x and MTK-A-x, mononuclear cells (MCs) were isolated from bone marrow or pleural effusion by gradient centrifugation at 800 g for 20 minutes at room temperature with brakes off using Lymphoprep according to standard protocols. Afterwards, MCs were washed once in PBS containing 2% FBS before resuspension in Matrigel diluted 1:2 with PBS to a total volume of 300 µL injected subcutaneously into the left flank of NOD./Cg-Prkdc^scid^Il2rg^tm1Wjl^/SzJ mice (NSG, Charles River, UK) mice at 6-8 weeks of age using a 30-gauge hypodermic AganiTM needle. To establish GR-ALCL-1, a 2-5 mm^3^ tumour lymph node paravertebral sample was implanted subcutaneously into both flanks of NSG mice at 6-8 weeks of age under anaesthesia (3% isoflurane, 1.5l/min air). According to UK Home Office animal licensing regulations and in accordance with the UKCCR guidelines, mice were euthanized once tumours reached 15 mm in any direction, due to reaching a humane endpoint other than tumour size or following 21 days of consecutive treatment, whichever was reached first.

### *In vivo* studies

For *in vivo* studies, PDXs at passage ≤4 and NSG mice at 6–8 weeks of age were employed in all experiments. Digital callipers were used to record tumour length and width daily, and tumour volumes were estimated using the modified ellipsoid formula $V=\frac{1}{2}ab^{2}$, where $a$ and $b$ are orthogonal tumour measurements and $a\geq b$.

### DNA Isolation and Whole Exome Sequencing

Single cell suspensions were obtained by mechanical disaggregation of excised tumours through a 70μm nylon cell strainer (Corning, UK) followed by 2 washes in PBS. Cells were collected by centrifugation at 200g for 5 minutes before lysis, and DNA extraction was performed using a DNeasy Bood & Tissue kit (Qiagen; cat. 69504). DNA quality and quantity was assessed by Nanodrop and Qubit (Thermo Scientific, UK). Library preparation and paired-end WES was conducted by Novogene (Cambridge, UK) for the MGS-A-x and MTK-A-x PDX, by the Genomics Core Facility (CEITEC, Masaryk University, Czech Republic) for diagnostic samples for patients 1 and 2, and by the INSERM U1015 and translational research unit (Institut Gustave Roussy, France) for samples from the patient and the resulting GR-ALCL-1 PDX^21^. Unfortunately, a relapse biopsy from patients 1 and 2 was not available to determine whether the established PDX preserves the genomic profile of the implanted tumour.

### Drug Dosing

### ALK inhibitor concentrations were inferred from the findings of previous *in vivo* studies carried out by the European Medicines Agency[^1^](#citeproc_bib_item_31). The crizotinib dose used converts to a human equivalent dose of approximately 300 mg/m^2^ using conversion formulas based on Freireich et al.[^2^](#citeproc_bib_item_32) based on a child weight of 20 kg and a mouse weight of 0.033 kg[^3^](#citeproc_bib_item_33). This dose is comparable to that used for some patients in the AcSé-Crizo (NCT02034981), COG-ANHL12P1 (NCT01979536) and COG-ADVL0912 (NCT00939770) trials.

### Bioinformatics

Pathologist-assessed tumour purity and that estimated by sequenza differed substantially (80% and 10% respectively; Supplementary Figure [1](#map006vafpath)). Sequenza estimated the tumour purity for the PDX tumour as 100%, which is consistent with the lack of engraftment of human stromal cells in mice, as well as successful *in silico* prefiltering of murine reads. Due to the potential for sampling variability between the material used to assess purity and the material from which DNA was extracted, the bioinformatically-estimated tumour purity was used to correct VAFs derived from the relapse biopsy (Figure [2](#engraftment_fig)e; see Figure S[1](#map006vafpath) for VAFs corrected by pathologist-assessed tumour purity).

### Immunohistochemistry

Tumours were fixed in 10% neutral-buffered formalin for 24 h, then paraffin-embedded and 3 µm sections cut from central regions. FFPE sections were stained with haematoxylin and eosin or with antibodies against CD30 and ALK. Immunohistochemistry was performed with the conventional avidin–biotin–peroxidase method. Heat antigen retrieval was performed using citrate buffer, pH 6.1 and endogenous peroxidases were quenched by incubating sections in 3% H_2_O_2_ in PBS for 10 minutes. First, sections were blocked using the Avidin/Biotin blocking kit. Then, the indicated primary antibodies were added in 1% BSA/PBS and incubated at 4°C overnight. Finally, slides were incubated with biotin-conjugated secondary antibodies using the IDtect Super Stain System – Horseradish peroxidase (HRP) and developed using the AEC substrate kit. Next, the sections were washed with PBS 3 times between each step. The stained slides were mounted with Aquatex and assessed by an experienced pathologist for both the intensity and percentage of positively stained cells. Positive staining was considered if present in >1% of cells. Corresponding diagnostic biopsy material was available for histology studies from Patients 1 and 2 but no relapse material was available for molecular characterisation.

### Resources and Reagents Sharing Statement

Please direct all requests for further information, resources and reagents to [sdt36@cam.ac.uk](mailto:sdt36@cam.ac.uk).

**Data Sharing Statement**

Custom scripts were used for data pre-processing, statistical analysis and data visualization. Please direct all requests for data and code to [sdt36@cam.ac.uk](mailto:sdt36@cam.ac.uk).

**References**

1. European Medicines Agency. Brigatinib Assessment Report. <https://www.ema.europa.eu/en/documents/assessment-report/alunbrig-epar-public-assessment-report_en.pdf> (2018).

2. Freireich, E., Gehan, E., Rall, D., Schmidt, L. H. & Skipper, H. Quantitative comparison of toxicity of anticancer agents in mouse, rat, hamster, dog, monkey, and man. Cancer chemotherapy reports (1966).

3. Guidance for Industry: Estimating the Maximum Safe Starting Dose in Initial Clinical Trials for Therapeutics in Adult Healthy Volunteers. (2005).

## Supplementary Tables

**Table S1: Reagents and resources**

| **Reagent or resource** | **Source** | **Identifier** |
| --- | --- | --- |
| ***Antibodies*** | | |
| Bond™ Ready-to-Use Primary Antibody ALK (5A4) | Leica Biosystems Newcastle Ltd | Cat#: PA0306 |
| BOND™ Ready-to-Use Primary Antibody CD30 (JCM182) | Leica Biosystems Newcastle Ltd | Cat#: PA0790 |
| ***Chemicals, Peptides, and Recombinant Proteins*** | | |
| Iscove's Modified Dulbecco's Medium (IMDM) | Thermo Fisher Scientific | Cat#: 21980032 |
| Penicillin/Streptomycin Solution | Thermo Fisher Scientific | Cat#: 15140122 |
| Fetal bovine serum (FBS) | Labtech | Cat#: FCS-SA/500 |
| Dubbecco's phosphate buffered saline (DPBS) | Sigma-Aldrich | Cat#: D8537 |
| Trypan blue | Thermo Fisher Scientific | Cat#: D8537 |
| Lymphoprep | Stem Cell Technologies | Cat#: 07801 |
| UltraPureTM LMP Agarose | Invitrogen | Cat#: 16520-050 |
| Dimethyl sulfoxide (DMSO) | Sigma-Aldrich | Cat#: D8418 |
| Crizotinib | Pfizer | N/A |
| Crizotinib | MedChemExpress | Cat#: HY-50878 |
| Brigatinib | MedChemExpress | Cat#: HY-12857 |
| Matrigel basement membrane matrix | Corning | Cat#: 354277 |
| IDetect Super Stain System – HRP | Empire Genomics | Cat#: IDST1007 |
| Target Retrieval Solution, Citrate pH6.1 (10X) | Agilent Dako | Cat#: S2369 |
| Aquatex | Merck | Cat#: 108562 |
| ***Critical Commercial Assays*** | | |
| RNeasy Plus Mini Kit | Qiagen | Cat#: 74134 |
| AEC Substrate Kit | BDPharmingen | Cat#: 551015 |
| Avidin/Biotin Blocking Kit | Vector Laboratories | Cat#: SP2001 |
| QIAquick PCR Purification Kit | Qiagen | Cat#: 28106 |
| ***Deposited Data*** | | |
| WES data of 2 ALK+ ALCL patient at diagnosis and 3 ALK+ ALCL PDX tumours | current study |  |
| **Experimental Models: Patient-derived xenografts** | | |
| MGS-A-x | current study | N/A |
| MTK-A-x | current study | N/A |
| GR-ALCL-1 | ^21^ | N/A |
| ***Experimental Models: Organisms/Strains*** | | |
| NOD.Cg-PrkdcscidIl2rgtm1Wjl (*Mus musculus*) | Charles River | Cat#: 005557; RRID: IMSR_ARC:NSG |
| ***Other*** | | |
| 2100 BioAnalyzer System | Agilent | N/A |
| 30-gauge hypodermic AganiTM needle | VWR | Cat#: 613-5373 |
| Plastic feeding tubes (22ga (black) x 38mm) | Instech Laboratories | Cat#: FTP-22-38 |
| 70 μM nylon Falcon™ cell strainer | Thermo Fisher Scientific | Cat#: 352350 |
| 5 mL Plastipak™ syringe | Thermo Fisher Scientific | Cat#: 302187 |
| High sensitivity D1000 ScreenTape | Agilent | N/A |

**Table S2: ALCL patient characteristics.** Characteristics of ALK+ ALCL patients from which samples were used to generate PDX. ALCL99 = cyclophosphamide, methotrexate, ifosfamide, etoposide, cytarabine, doxorubicin; ALCL99* = patient was treated according to ALCL99 recommendations for patients with central nervous system involvement; CYVE = cytarabine, etoposide; Event-free survival (EFS) was defined as the time to recurrence or relapse. Overall survival (OS) was determined as cancer-specific death. Dx = diagnosis, N/A = not applicable, CT = chemotherapy, r/r = relapsed/refractory, IV = intravenous, SCT = stem cell transplant, nd = not determined.

| **Characteristics** | **Patient 1** | **Patient 2** | **Patient 3** |
| --- | --- | --- | --- |
| ***Clinical characteristics*** | | | |
| **Sex** | F | M | M |
| **Age at dx** | 6 years | 5 years | 16 years |
| **CNS involvement** |  |  |  |
| at dx | No | No | Yes |
| at CT r/r | Yes | No | unknown |
| at crizotinib r/r | Yes | Yes | unknown |
| **Time to 1st relapse** | 4 months | 1 month | unknown |
| **Time to death** | 13 months | 6.5 months | N/A |
| ***Treatment protocols*** | | | |
| **1st-line** | ALCL99 | ALCL99 | ALCL99* |
| **2nd-line** | Vinblastine | CYVE | Crizotinib |
| **3rd-line** | Vinblastine | Crizotinib | Nivolumab |
|  | IV CYVE  intrathecal CT | intrathecal CT |  |
| **4th-line** | Vinblastine Crizotinib intrathecal CT | CYVE | N/A |
| **5th-line** | SCT | Vinblastine steroids | N/A |
| **6th-line** | Crizotinib | N/A |  |
| **7th-line** | Brentuximab Vedotin vinblastine | N/A |  |
| ***PDX characteristics*** | | | |
| **Name** | MGS-A-x | MTK-A-x | GR-ALCL-1^21^ |
| **Source of tumour cells** | Bone marrow | Pleural effusion | Paravertebral lymph node |
| ***ALK mutation status*** | | | |
| at diagnosis | nd | nd | nd |
| at CT r/r | nd | ALK wildtype | nd |
| at crizotinib r/r | ALK C1156Y | nd | ALK wildtype |

## Supplementary Figures


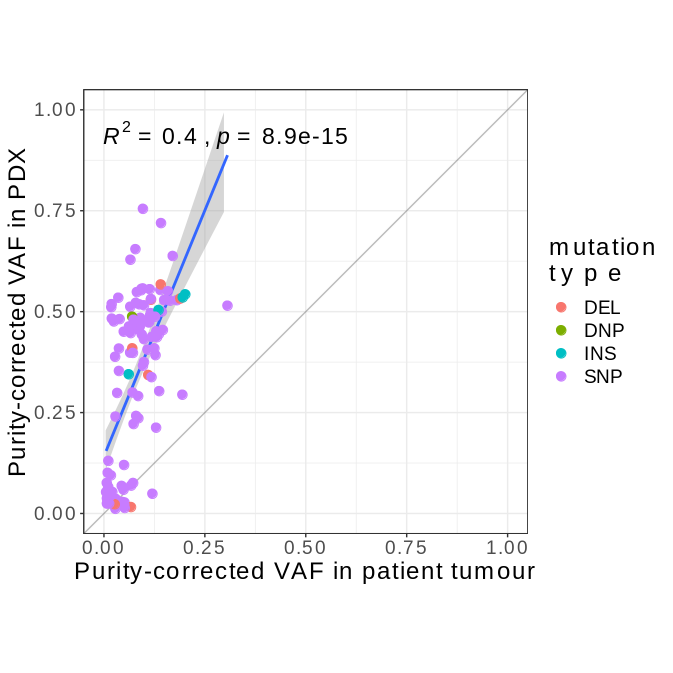


**Figure S1:** Data corrected for purity according to pathologist-assessed tumour purity. DEL = deletion, INS = insertion, SNP = single nucleotide polymorphism, DNP = dinucleotide polymorphism, PDX = patient-derived xenograft, VAF = variant allele frequency.


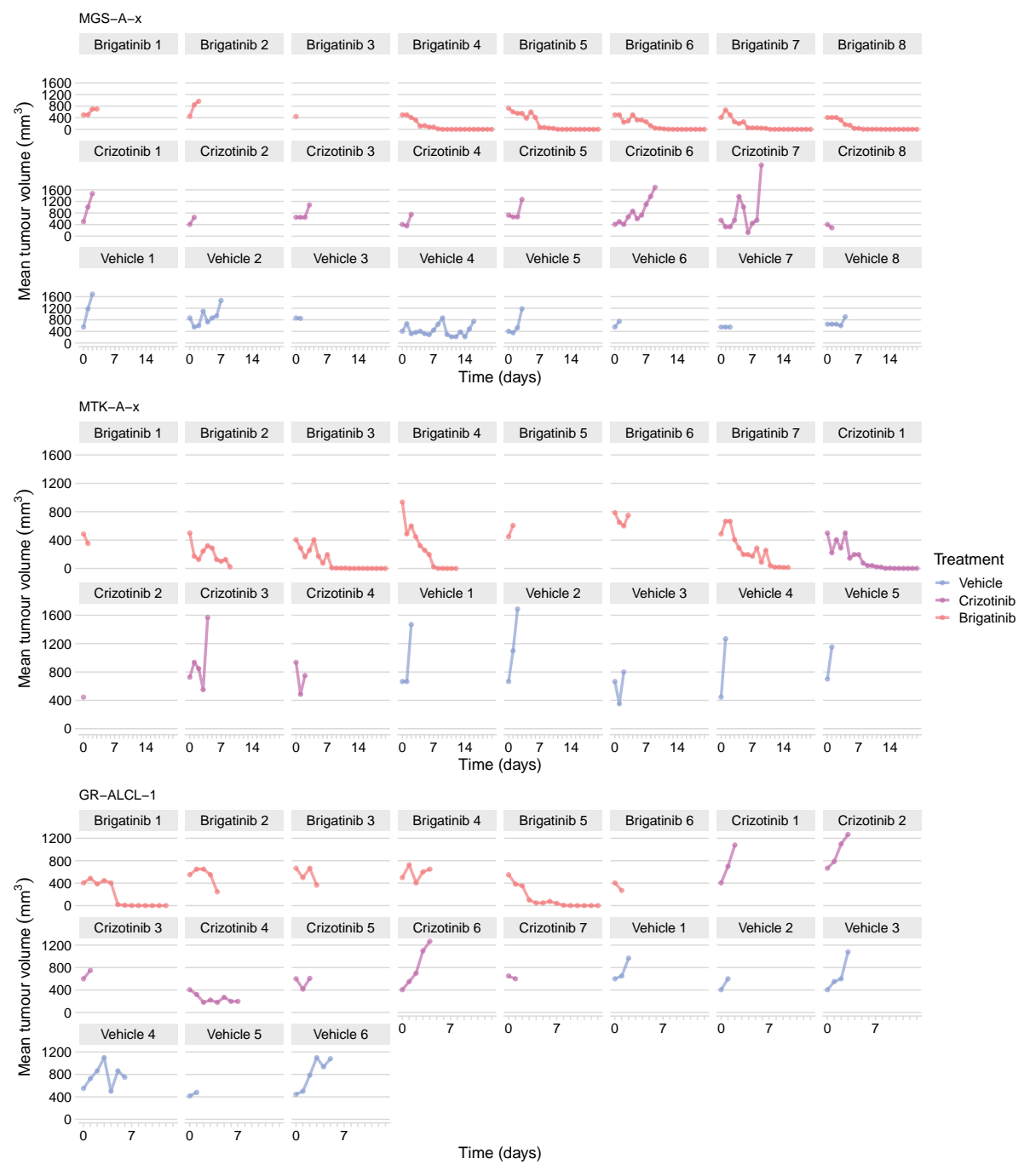


**Figure S2:** Tumour volume over time for individual mice bearing MGS-A-x (top graphs), MTK-A-x (middle graphs), or GR-ALCL-1 (lower graphs) PDX tumours, treated with crizotinib, brigatinib, or vehicle only.


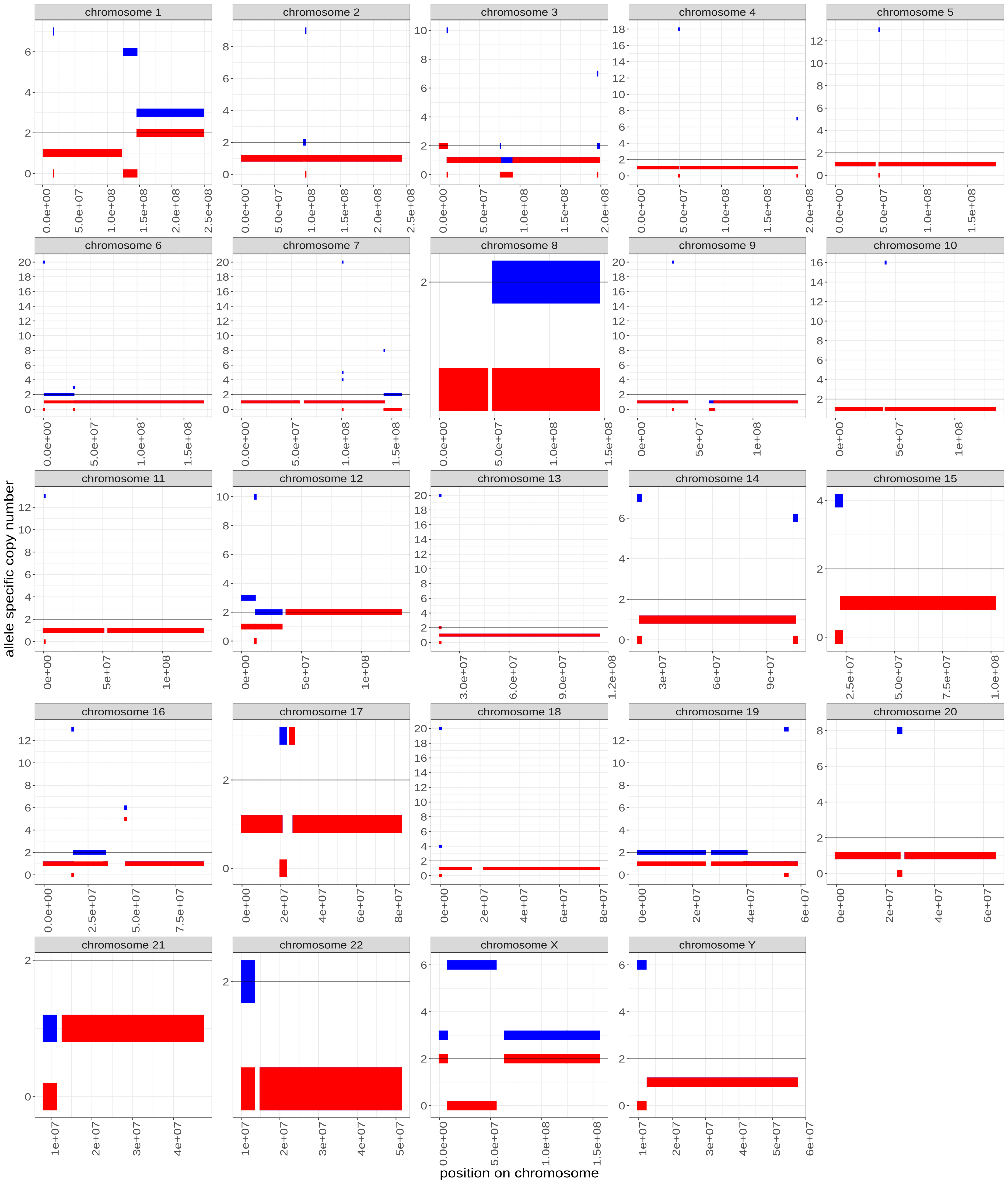


**Figure S3:** Genome-wide allele-specific copy number for the relapse-derived sample from patient 3 presented according to chromosomes.
